# Has knowledge of the vaginal microbiome altered approaches to health and disease?

**DOI:** 10.12688/f1000research.13706.1

**Published:** 2018-04-13

**Authors:** Gregor Reid

**Affiliations:** 1 Departments of Microbiology & Immunology, and Surgery (Urology), Western University, and Lawson Health Research Institute, 268 Grosvenor Street, London, ON, N6A 4V2, Canada

**Keywords:** vaginal, microbiota, microbiome, Lactobacillus, health, disease

## Abstract

Sixteen years ago, when we published the first molecular characterization of the vaginal microbiota, little did we know the vast numbers of species that would be detected in this niche. As exciting as these discoveries have been, what have they and more recent advances contributed to how vaginal health and disease are managed? This review provides a brief discussion of the potential, but so far limited, applications that have arisen from microbiome research. Calls for innovation have been made before but to little avail.

## Introduction

The anatomical location and function of the vagina make it potentially affected by many internal and external factors. These include hormonal changes, menstruation, the gastrointestinal microbiota at the nearby rectum, the act of bathing and products used for hygiene, sexual interactions, and use of various contraceptives. Although clinical emphasis often is placed on aberrant conditions, such as bacterial vaginosis (BV), sexually transmitted infections, and cancer, it is quite remarkable that the niche is, for the most part, healthy and recalcitrant to any aberrations ^1^.

The cleansing action of cell turnover, mucus production, and local immune defenses all play a role in maintaining homeostasis ^2^, but it is the role of beneficial bacteria that arguably has drawn most scientific interest ^3– 6^.

Even with known limitations in molecular methodologies, numerous studies have described the detection of several hundred bacterial types in the vagina of healthy and non-healthy subjects in countries around the world ^7– 11^. The consistent dominance of only a handful of *Lactobacillus* species is quite remarkable and likely of evolutionary importance to vaginal health and human reproduction ^1, 12^. These microbiome studies, and others performed with samples from multiple human sites, have dramatically changed the way the human body is perceived. It was simpler to think of microbes only for being the cause of disease. Now, we must decipher whether many of them are passengers or symbionts or are present to perform specific functions that promote and maintain health. Even this is overly simplistic, as it turns out that the reactions are likely a result of the collective microbiota rather than of an individual species. This is quite different from the pathogenesis perspective centered on Koch’s postulates.

## The dilemmas we now face

The definition of normal has proven as elusive as the definition of BV ^13^ given that bacteria present in BV can also be present, and even dominate, in healthy women. An unanswered question is whether a portion of healthy women colonized by BV organisms have an increased susceptibility to subsequent pathology, including pregnancy complications and increased risk of acquiring sexually transmitted infections and symptomatic BV itself.

In assessing whether an individual is ill or healthy, there has to be something to test. This presents two clinical dilemmas. One is the sparsity of point-of-care tests that can be used by clinicians when a symptomatic patient visiting a clinic has unusual vaginal discharge or genital itching or both. Physical examination can help to differentiate BV, vulvovaginal candidiasis (VVC), and urinary tract infection (UTI), and the findings of discharge, abnormal pH and Pap smears, lesions, warts, and inflammation are all helpful for diagnosis. If feasible on site, microscopic assessment of vaginal epithelial cells is somewhat useful. The second dilemma is that many women with BV, VVC, UTI, herpes simplex recurrences, or even trichomoniasis are asymptomatic ^14^. Therefore, they will not seek care and could have complications of these infections. This raises a question of whether, and how often, women should be encouraged to be screened for these infections even if they have no symptoms. The answer may also depend on the insurance coverage for costs associated with screening. For pregnant women, testing for asymptomatic infections could make sense, but what happens if the diagnosis is positive?

Curable sexually transmitted infections should always be treated to prevent complications and to break the chain of transmission. However, for asymptomatic BV and UTI, antibiotic treatment is not normally prescribed, nor does it necessarily prevent recurrences. So this presents another dilemma for the physician. Both of these conditions could increase the risk of premature labor ^15^, and antibiotics themselves may be detrimental to the fetus ^16^. Plus, the conditions often resolve on their own.

All this assumes that the screening tools are good and practical. The Gram stain Nugent Score made a valiant attempt to identify an intermediary state, in which lactobacilli and pathogens were present ^17^. But it is only observational at best and does not inform clinicians how to approach patient management. So far, immunological approaches have been close to useless in terms of novel insight into what the vaginal microbiome does and the practical applications thereof. Discharge can come in different forms or be absent, and no practical system has been developed to measure, differentiate, and interpret cytokines, chemokines, T cells, sIgA, or other immune parameters. Indeed, the role of inflammation per se is controversial, even for BV ^18^.

A multiplex polymerase chain reaction test can differentiate organisms responsible for BV, VVC, and UTI, but few office settings have this in place. Rapid microbiota testing is still not available or affordable, but if the microbiota patterns were truly definitive, how would these be interpreted? The presence and abundance of certain *Lactobacillus* species over lesser detection of other species could indicate a healthy vaginal status, but their absence need not mean an unhealthy condition. For asymptomatic patients, such a result may confirm good health and lead to a decrease in the over-prescription of antibiotics.

For patients with signs or symptoms of various degrees of discomfort, discharge, itching, pain, dryness, and odor, what do these microbiota tests add to existing knowledge? The answer is not clear, nor is the best treatment.

A major problem has been the paucity and poor efficacy of interventions. Essentially, two classes of antibiotics and not many more types of anti-fungals represent clinical treatment options. The inability to penetrate and eradicate microbes in biofilms, target efficiently only the organisms causing harm, and then promote recovery of the beneficial microbes illustrates the huge limitations of these options. In fact, they represent an incredible lack of innovation among the pharmaceutical industry and science community given the massive number of women who potentially need effective products. A literature scan of clinical trials suggests that a vaginal ring matrix of ethylene vinyl acetate and methacrylic acid-methyl methacrylate copolymer loaded with 150 mg DL-lactic acid represents an attempt to develop a new prophylactic therapy ^19^. Three other approaches are probiotics (to be discussed later), estrogen-containing contraceptives, such as the NuvaRing, which helps improve the vaginal microbiota (manuscript by Tania Crucitti *et al*. is in press), and a BufferGel to reduce BV prevalence ^20^.

## Alternative therapies

Since vaginal and urinary issues severely impact quality of life, women have turned to alternative therapies, including douching, probiotics, steam baths, holistic sexology, cranberry and lemon juice, and an array of so-called traditional practices to try to find relief ^21– 26^. Some of these products make claims that sound like they are based in microbiome science (for example, “At Daengki Spa in Koreatown, proprietors claim the treatment will ‘rid the body of toxins’ and help women with menstrual cramps, bladder infections, kidney problems and fertility issues” ^27^). However, the lack of efficacy data illustrates their fallacy.

Of these alternatives, probiotics warrant the most attention. The concept of their application was to use species that could compete against offending pathogens or disease processes or species normally highly abundant at that site that could replenish low abundances of existing lactobacilli, thereby allowing indigenous ones to return. Two delivery approaches have been considered: directly into the vagina and oral administration. The advantage of the former is that the lactobacilli are delivered to the problem area where their activity can target pathogens more quickly. A disadvantage is that this represents a drug treatment, according to regulatory authorities, and therefore the expenditure is prohibitive to academic researchers and many companies. The oral route ostensibly simulates the natural means by which pathogens transfer from the rectum to the vaginal area because of anatomical proximity. This is believed to induce microbial competition, thereby reducing pathogen ascension and infection recurrence. The disadvantage is that the time for this to occur is longer than direct administration into the vagina and the billion or so lactobacilli ingested have to compete with trillions of microbes in the gastrointestinal tract and then make their way to the vagina. The net result is that relatively few lactobacilli ingested reach the vagina. Nevertheless, the treatment still causes an increase in total lactobacilli in the vagina ^28^.

Despite limited funding for human studies, there is evidence to suggest that probiotics contribute toward disease treatment and prevention ^29, 30^. Probiotic lactobacilli do not induce inflammation, drug resistance, or other detrimental effects, making them worthy of clinical consideration for the treatment of BV and the prevention of BV, VVC, and UTI.

Admittedly, these approaches precede the microbiome era, but knowledge of the vaginal microbiome has not altered the strategy. Rather, probiotics were ahead of their time. The identification of *L. iners* in the vagina again pre-dates the microbiome era ^31^, although its prevalence was subsequently better appreciated with 16s rRNA datasets. Notably, this has not led to its being used as a probiotic, in part because it has fastidious growth requirements ^32^ and because it appears to depend on which clone can confer health benefits ^33^. Probiotic strains *Lactobacillus rhamnosus* GR-1 and *Lactobacillus reuteri* RC-14 were developed long before the microbiome era ^30^ and are administered orally as supplements in around 40 countries and as vaginal suppositories in a few. They fit into the criteria of species not so commonly abundant in the vagina but strains that are delivering activities to offset aberrant conditions. The clinical development of *Lactobacillus crispatus* CTV-05 (product name Lactin V) originated from its ability to produce hydrogen peroxide and being a common species in the vagina ^34^. Studies have shown that *L. crispatus* CTV-05 colonized the vagina of women whose indigenous lactobacilli were absent ^35^ and in doing so could reduce the recurrence of UTI ^36^.

Too often, probiotic formulations appear to be based upon strains that large companies make available or that have already been approved as probiotics or strains whose species have been found in the vagina. Instead, probiotic strains should be selected for specific attributes and activities that contribute to what indigenous lactobacilli do or what is deficient in the host. In the case of *L. crispatus* CTV-05, it is difficult to imagine that hydrogen peroxide production is its primary attribute, as the extent to which this compound protects the host from infection, or indeed maintains a healthy niche, has not been definitively proven.

It could be argued that diseases, such as BV, caused by communities of microbes require treatment that disrupts these communities ^37^. Whether this will require enzymes that degrade biofilm glycocalyx, upregulate host antimicrobial activity, or induce reactions inside the microbes that turn off virulence genes remains to be seen. *In vitro* studies have suggested that this may be possible by applying single strains of probiotic lactobacilli that penetrate and disrupt biofilms ^38^.

Given the success of total fecal replacement therapy (fecal microbiota transplant) in treating *Clostridium difficile* infection, the idea of a vaginal microbiota transplant (VMT) is worth considering if all other therapeutic options have failed. This will pose ethical questions and create practical issues of whose VMT should be used. With no suitable animal models, such a concept would have to be tested in humans. It may be that a *L. crispatus*-dominant VMT is applied to women whose most abundant species in a healthy state was *L. crispatus*, but the same VMT may not work for women who had *L. iners*, *L. gasseri*, or *L. jensensii* or another as the majority species. The size of inoculum may prove a problem if sufficient numbers of bacteria are not extracted from the donor. Immunological assessment will also be required, as antigens from the donor will be present in the material. In *C. difficile* cases, potent antibiotics are used to deplete the recipient’s microbiota. Presumably, this would be required prior to VMT, although use of these agents seems counter-intuitive.

If and when VMT and other applications are tested, we might be able to acknowledge the role of microbiome research in patient care. Unfortunately, such a situation does not seem to be in the near future. This concept may be precipitated if Lactin V phase III results are disappointing to prevent BV or UTI recurrences or both. If that happens, it is not clear from a scientific perspective what other components of a VMT would be more apt to work than *L. crispatus*. The one issue worth pointing out is that the CTV-05 application is given after antibiotics. Given the disruption to the microbiota caused by these drugs, a reason for failure (if it occurs) might ironically be the antibiotics eradicating other organisms needed by *L. crispatus* for it to re-establish a healthy state.

In the intestinal tract, it was thought that the host’s microbiome had to be radically altered by probiotic strains before homeostasis could be restored. Thus, when the probiotic strains were found not to colonize, it led to negative perceptions about these therapies. In fact, probiotic colonization or major alteration in the indigenous microbiota may not be critical for restoration of a healthy state in many cases. Health may be conferred as long as the probiotic strains are being ingested and altering the metabolic output. Potentially, the same could be said for the use of prebiotics in the vagina. A recent study suggested that lactulose could manipulate the microbiome, potentially helping to restore homeostasis ^39^.

Any use of VMT would require studies to investigate whether negative long-term outcomes emerge that, for example, put the recipient at risk of a disease other than the one that was being treated.

Another problem is the ineptitude of the regulatory systems in Europe and the United States in terms of still requiring any treatment or prevention of a disease to follow a drug route that historically has comprised chemicals. A system that denies that nutrition is important for vaginal health or that regards reinserting non-pathogenic *Lactobacillus* into a niche where they are naturally found as the equivalent to applying a potent, destructive chemical like an antibiotic to the same niche ^40^ really needs an overhaul.

If true innovation is to be developed, tested, and applied, new directives need to be given to regulatory agencies to allow for the application of microbes to niches from which they have long since originated. This is not to suggest that safety is no longer paramount. Rather, it is to acknowledge that humans exist because of microbes in a microbial world, and the sooner we implement change across societal and governmental agencies to acknowledge this, the sooner we will identify means to use and manipulate microbes for the betterment of human and planetary health. The status quo, originated because of people selling snake oil over a hundred years ago, is no longer rational. Most importantly, the health of women requires that we explore all possible interventions to allow their quality of life to improve and chronic suffering to decline.
